# Knowledge, attitude, and perception of the parents toward HPV vaccine administration to their children in Saudi Arabia: a cross-sectional study

**DOI:** 10.3389/fpubh.2025.1531517

**Published:** 2025-07-16

**Authors:** Abubaker S. Bakhashab, Saja A. Aljilani, Noura M. Alkinaidri, Abdulaziz A. Felimban, Moheealdeen H. Habbal, Nawaf A. Bashah, Ragia H. Ghoneim, Diena Almasri, Razan K. Thabit, Masaad Saeed Almutairi, Abrar K. Thabit

**Affiliations:** ^1^Faculty of Pharmacy, King Abdulaziz University, Jeddah, Saudi Arabia; ^2^Department of Pharmacy Practice, Faculty of Pharmacy, King Abdulaziz University, Jeddah, Saudi Arabia; ^3^King Fahad General Hospital, Jeddah, Saudi Arabia; ^4^Department of Pharmacy Practice, College of Pharmacy, Qassim University, Qassim, Saudi Arabia

**Keywords:** human papillomavirus virus, vaccine, hesitancy, papillomavirus infections, papillomavirus vaccines, vaccination hesitancy, perception, Saudi Arabia

## Abstract

**Background:**

Human papillomavirus (HPV) infection is a major culprit of infection-related cancer globally. In Saudi Arabia, HPV vaccine was recently introduced. We assessed the knowledge, attitude, and perception of parents in Saudi Arabia toward HPV vaccine uptake.

**Methods:**

In this survey-based cross-sectional study, eligible participants (parents of girls or boys aged 9–14 years) were interviewed in-person. The survey was divided into sections: demographics, HPV infection and HPV vaccination knowledge, vaccine acceptance in general, and HPV vaccine acceptance.

**Results:**

Of 386 participants, 65.5% were fathers, 44.6% were aged 30–39 years, and 78% held at least a college degree. Knowledge regarding HPV risk and its transmission was overall low. While 64% have heard of HPV infection, 56% expressed their willingness to vaccinate their children against it. Perception of HPV infection risk to others was the only factor associated with HPV vaccine uptake acceptability (OR, 2.49; 95%CI, 1.37–4.52; *p* = 0.003). Lack of information (15.1%) and fear of side effects (13.6%) were stated by participants unwilling to vaccinate their children. Social media/internet was the major information source for those who had heard of HPV. However, many participants wanted to learn about HPV from healthcare providers.

**Conclusion:**

This study showed lack of knowledge by the public in Saudi Arabia regarding HPV infection and its associated risks, which may have been associated with hesitancy to vaccinate their children. Healthcare providers are encouraged to educate their patients and the public about HPV and the importance of the vaccine in media outlets and in their practice.

## Graphical abstract

Factors influencing acceptability of parents of HPV vaccine administration to children.
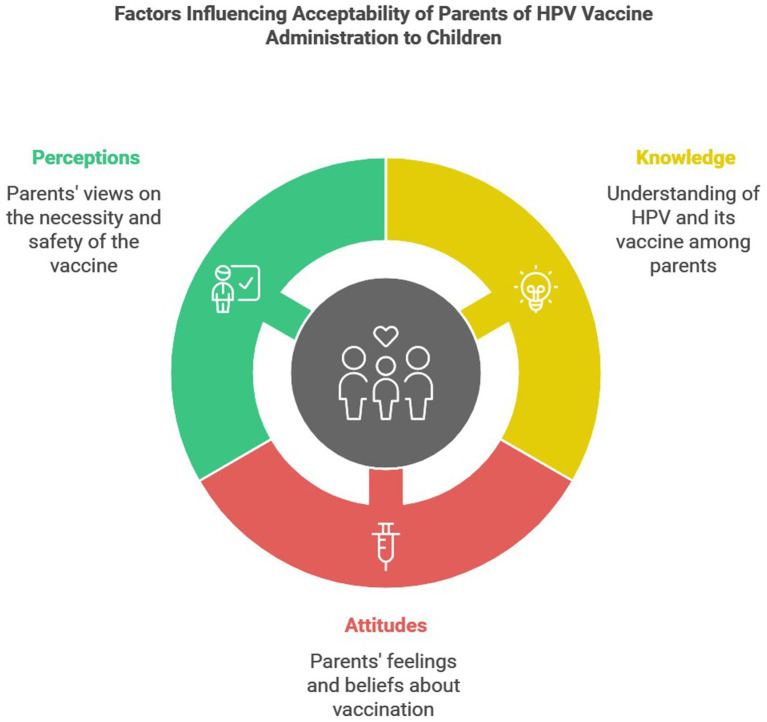

## 1 Introduction

Human papillomavirus (HPV) is a highly prevalent virus with more than 40 types transmitted sexually between adults. HPV infections could be asymptomatic infections; however, certain types could result in genital warts, while others could persist within the squamous cells and transform into malignancy in different organs, such as cervix, ovaries, vagina, anus, penis, and oropharynx (1, 2).

Globally, the estimated prevalence of HPV infection is 11.7% in a meta-analysis of over a million women with normal cervical cytology (3). In Saudi Arabia, HPV infection prevalence ranged between 4.7 and 30.4% during the period from 2013 to 2021 (4). Additionally, one study from Saudi Arabia found that 77% of women with cervical cancer were originally infected with HPV (5). Among women with cytological cervical abnormalities, the prevalence of HPV detection was 52.9% in a study that was conducted between 2021 and 2022 (6). According to a recent report from 2023, it is estimated that more than 10 million women in Saudi Arabia are at risk of HPV infection with a cervical cancer incidence rate of 2.44 per 100,000 woman (7). While the reported incidence rate of HPV infection in Saudi Arabia appears to be relatively low, prevention strategies are crucial for mitigating the transmission of the virus, and consequently reducing the burden of HPV-associated malignancies that are linked to increased morbidity, reduced quality of life with the use of chemotherapy, and perhaps increased mortality if undiagnosed timely (8, 9). Unfortunately, most Saudi females infected with HPV visit hospitals at an advanced stage. This could be attributed to several factors, including lack of awareness, knowledge, follow up screening recommendations, organized regional campaigns for HPV vaccine, and health promotion programs, which are currently heavily focused on other chronic diseases or common malignancies (such as breast cancer, diabetes, and obesity) (10). To improve the screening and early management, the Saudi Ministry of Health (MOH) published HPV screening practice guidelines in 2014 (11); however, there is no national screening program for HPV and cervical cancer yet (12, 13).

To combat the spread of HPV and consequently control the development of associated cancers, HPV vaccine was developed two decades ago. The first HPV vaccine was approved by the US Food and Drug Administration in 2006. However, the vaccines that are commonly used nowadays are those that cover the carcinogenic types (HPV16 and HPV18) (8). One vaccine covers only the two carcinogenic types (Cervarix^®^), whereas the other vaccine covers both of these types in addition to seven other types that are associated with warts (Gardasil9^®^). The vaccines are approved for both males and females from as early as 9 years. HPV vaccines could decrease the mortality rates among low-to-middle income women over the next decades according to World Health Organization (WHO) when administered to up to 90% of the targeted population of girls by the age of 15 (9). The first HPV vaccine was approved by the Saudi Food and Drug Authority in 2008 (14). Though, Cervarix^®^ has been available in Saudi Arabia since 2012, while Gardasil9^®^ has been available since 2024, both for females of the age range 9–45 years (15–17). Though, it was only recently widely administered in 2017 when the Saudi MOH included it in the national childhood immunization schedule for girls aged 11–12 years at no cost (18). Unfortunately, previous reports from Saudi Arabia found that most women, including students in health fields, were unaware of the HPV vaccine availability (10, 19–21).

We noticed that previous studies that evaluated the acceptability of HPV vaccine in Saudi Arabia uptake only included adult women (19–27). However, since the inclusion of the vaccine in the Saudi national immunization schedule in an effort to expand its administration among girls in Saudi Arabia, very few studies from Saudi Arabia have evaluated the acceptability of parents of children (both girls and boys) aged 9–14 years to administer the vaccine to their children. Though, most of these studies either did not include a sufficient sample size to achieve a high power or distributed the survey via online platforms risking multiple biases, such as selection bias and non-response bias. Therefore, this study aimed to evaluate HPV infection knowledge and HPV vaccine acceptability by parents in Saudi Arabia via in-person interview after calculating the required sample size to achieve high power. We also aimed to evaluate the impact of different sociodemographic factors and knowledge on the willingness to administer HPV vaccine to the parents’ children.

## 2 Methods

### 2.1 Study design and population

We conducted a cross-sectional survey-based study via in-person interviews from November 2023 to August 2024. Eligible participants (i.e., the study unit) were individual parents (either the mother or the father) who had at least one child, a girl or a boy, within the age range of 9–14 years as this is the age range specified for the childhood dosing schedule of HPV vaccine (9). The participants were recruited using purposive sampling at waiting areas of pediatric clinics and public spaces, such as playgrounds of parks and shopping malls. Only Saudi citizens and legal residents were included; thus, temporary visitors to Saudi Arabia were excluded. No disabled participants were included in the study during the recruitment period. The participants were asked to provide verbal consent before taking part in the study. The ethical approval was obtained from the Research Ethics Committee of the Faculty of Pharmacy, King Abdulaziz University, Jeddah, Saudi Arabia (reference no. PH-1445-8).

### 2.2 Questionnaire development

The questions of the questionnaire were adapted from a previously published study (28), where the questions were translated to Arabic. The survey was divided into five sections to gather data on sociodemographic traits, HPV vaccination knowledge, vaccine uptake acceptance in general, knowledge of HPV, and acceptability of HPV vaccine uptake. A brief pilot phase with six participants preceded data collection to ensure the clarity of the survey questions and to validate the accuracy of their Arabic translation. Additionally, the source questionnaire was reviewed to ensure the absence of culturally inappropriate items. Since some questions were amended for clarity, results from the pilot phase were not included in the final analysis. Additionally, a test–retest reliability assessment of the questionnaire was conducted again with the same six participants a few weeks later to ensure the consistency of their responses. Furthermore, some questions were deleted that were deemed irrelevant and to shorten the length of the survey, such as listing the ages of all the children in the household and the length of time the person has held the local nationality. The interview methodology was employed in part to enhance the clarity of the survey instrument and to provide clarification for any questions that were not readily understood by participants, thereby mitigating potential response bias due to misinterpretation. An English version of the questionnaire utilized in this study is available in the Supplementary material. Reliability testing using Cronbach’s Alpha was not calculated as the survey questions were not dichotomous.

### 2.3 Sample size calculation

The current estimated population size of Saudi Arabia is approximately 30 million. In order to meet a 95% confidence level with a 5% margin of error, the total number of participants needed was 385. Qualtrics^®^ sample size calculator was used to calculate the sample size (29).

### 2.4 Statistical analysis

At the end of data collection, data were coded on Microsoft Excel (Microsoft Corp., Seattle, WA, United States) and later analyzed on SPSS version 24.0 (IBM Corp., Armonk, NY, USA). Results are presented using frequencies and percentages. To evaluate the factors associated with HPV vaccine uptake acceptability, certain variables of interest were analyzed using multivariable logistic regression to calculate odds ratios (OR) and 95% confidence intervals (95% CI). The significance of the model was established using the omnibus test of model coefficients. Additionally, the goodness-of-fit of the model was established using Hosmer-Lemeshow test. A *p-*value < 0.05 was deemed significant.

## 3 Results

### 3.1 Sociodemographic traits of the participants

A total of 386 participants agreed to take part in the study. Table 1 enlists the sociodemographic traits of the interviewed participants. Most respondents were fathers (65.5%), followed by mothers (31.6%), with a lower percentage of other relatives. Among the participants, 44.6% were between 30 and 39 years old and 29.8% were between 40 and 49 years old. More three-quarters of the population had at least an undergraduate college degree and were employed. In terms of social status, most participants were married (94.6%), and 81.8% had at least two children. When asked about the history of HPV vaccine uptake by the children, 79.8% said no. Approximately a similar number of participants also reported not receiving the HPV vaccine themselves (84.5%).

**Table 1:** Sociodemographic characteristics of the participants (*n* = 386).

Characteristic	*N* (%)
Relationship	
Mother	122 (31.6)
Father	253 (65.5)
Sibling	2 (0.5)
Grandmother	2 (0.5)
Aunt	4 (1)
Uncle	3 (0.8)
Age (years)	8.27 ± 5.26
<29	43 (11.1)
30–39	172 (44.6)
40–49	115 (29.8)
50–59	49 (12.7)
≥60	7 (1.8)
Education	
Elementary	6 (1.6)
Middle school	9 (2.3)
High school	70 (18.1)
Undergraduate	245 (63.5)
Postgraduate	56 (14.5)
Employment	
Employed	296 (76.7)
Housewife	39 (10.1)
Retired	25 (6.5)
Unemployed	22 (5.7)
Student	4 (1.0)
Nationality	
Saudi	373 (96.6)
Non-Saudi	13 (3.4)
Social Status	
Married	365 (94.6)
Divorced	14 (3.6)
Widowed	7 (1.8)
Number of children	
1	67 (17.4)
2	101 (26.2)
3	80 (20.7)
4 or more	138 (35.8)
Child gender	
Male	203 (52.6)
Female	183 (47.4)
Child received HPV vaccine	
No	308 (79.8)
Yes	33 (8.5)
Unsure	45 (11.7)
Parent received HPV vaccine	
No	326 (84.5)
Yes	25 (6.5)
Unsure	35 (9.1)

HPV, human papilloma virus.

### 3.2 Knowledge of HPV

As shown in Table 2, only more than one-third of the participants (36%) have not heard of HPV. Among the 64% who have heard of HPV, social media and the internet were the major sources of information (17.6%). More than half of the participants (53.4%) were unsure of HPV mode of transmission compared with only 32.4% who correctly identified it as a sexually transmitted infection. Despite the overall relatively low awareness rate of HPV, approximately half of the participants (49%) indicated that women are the most susceptible to the disease, followed by girls (27.7%), but 33.7% were unsure of the answer. Regarding the diseases caused by HPV, about half of the participants (49.5%) expressed having insufficient information. Nonetheless, 32.4 and 18.9% mentioned cervical cancer and genital warts as some of the most important conditions associated with HPV infection. Prevention of HPV acquisition by HPV vaccine uptake was indicated by 51.6%, while 24.4 and 20.7% said that it could be prevented by abstention from sexual intercourse and condoms, respectively.

**Table 2:** The knowledge of the participants regarding human papilloma virus (*n* = 386).

Question	*N* (%)
Have you heard of HPV?	
Yes	247 (64)
No	139 (36)
From where you got information about HPV?^*^ (*n* = 141)	
Social media and internet	68 (17.6)
Family or friends	22 (5.7)
Family medicine physician	18 (4.7)
Other healthcare provider	16 (4.1)
School	13 (3.4)
TV or radio	13 (3.4)
Gynecologist	10 (2.6)
Pediatrician	8 (2.1)
Pharmacist	9 (2.3)
Newspaper	4 (1)
How does HPV spread in your opinion?^*^	
Sexually	125 (32.4)
Blood	70 (18.1)
Mother to child	52 (13.5)
Toilets	42 (10.9)
Unsure	206 (53.4)
Is HPV the most common sexually transmitted infection in your opinion?	
Strongly agree	31 (8)
Agree	60 (15.5)
Disagree	57 (14.8)
Highly disagree	23 (6)
I do not know	215 (55.7)
Is HPV considered one of the most dangerous diseases in your opinion?	
Strongly agree	58 (15)
Agree	119 (30.8)
Disagree	27 (7)
Strongly disagree	7 (1.8)
I do not know	175 (45.3)
What is the category most susceptible in your opinion?^*^	
Women	189 (49)
Girls	107 (27.7)
Men	73 (18.9)
Boys	47 (12.2)
Unsure	130 (33.7)
What diseases are most related to HPV in your opinion?^*^	
Cervical cancer	125 (32.4)
Vaginal cancer	85 (22)
Genital warts	73 (18.9)
HIV	58 (15)
Valvular cancer	45 (11.7)
Anal cancer	43 (11.1)
Penile cancer	45 (11.7)
Bladder cancer	39 (10.1)
Oral cancer	23 (6)
Recurrent cystitis	21 (5.4)
Hepatitis	20 (5.2)
Infertility	20 (5.2)
Esophageal cancer	13 (3.4)
Irritable bowel syndrome	6 (1.6)
None	5 (1.3)
Not enough information	191 (49.5)
In your opinion what are the measures that can be done to prevent transmission of HPV?^*^	
HPV vaccine	199 (51.6)
Abstain from sexual intercourse	94 (24.4)
Personal hygiene	82 (21.2)
Condom	80 (20.7)
Antibiotics	18 (4.7)
Cannot be prevented	6 (1.6)
I do not know	119 (30.8)

HIV, human immunodeficiency virus; HPV, human papilloma virus.

*Some participants selected more than one answer.

### 3.3 General knowledge and acceptability of vaccines uptake

The vast majority of the respondents (88.1%) either agreed or strongly agreed that vaccines are effective against common infectious diseases in children and adults, while only a small fraction (4.4%) believed that vaccines are not effective (Table 3). Regarding the risk–benefit perception, 80.8% of the respondents believed that the benefits of vaccines outweigh their risks versus 9% who thought otherwise, and 10.1% were unsure. When it comes to the belief in vaccine harm, 84.7% of the respondents rejected the idea that vaccines are harmful; though, a minority of 7.5 and 7.8% believed that vaccines might be harmful or were unsure, respectively. The perception of herd immunity was also explored, where the notion that an unvaccinated child can put other children at risk was thought to be true by most respondents (79.3%) compared with 13.2% who did not believe in this risk, and 7.5% were unsure. Consequently, most respondents (72.8%) were unafraid to vaccinate their children, while 22% expressed concerns regarding childhood vaccination.

**Table 3:** General knowledge of vaccines and acceptability of vaccine uptake (*n* = 386).

Question	*N* (%)
Generally, vaccines are effective against common diseases in children and adults	
Strongly agree	162 (42)
Agree	178 (46.1)
Disagree	13 (3.4)
Strongly disagree	4 (1)
I do not know	29 (7.5)
Generally, vaccine benefits out way the risks	
Strongly agree	134 (34.7)
Agree	178 (46.1)
Disagree	31 (8)
Strongly disagree	4 (1)
I do not know	39 (10.1)
Generally, vaccines are harmful	
Strongly agree	10 (2.6)
Agree	19 (4.9)
Disagree	175 (45.3)
Strongly disagree	152 (39.4)
I do not know	30 (7.8)
If your child remained unvaccinated, this would put other children at risk	
Strongly agree	130 (33.7)
Agree	176 (45.6)
Disagree	43 (11.1)
Strongly disagree	8 (2.1)
I do not know	29 (7.5)
Are you afraid of vaccinating your child?	
Strongly agree	20 (5.2)
Agree	65 (16.8)
Disagree	163 (42.2)
Strongly disagree	118 (30.6)
I do not know	20 (5.2)

### 3.4 Knowledge of HPV vaccine

Table 4 shows that almost two-thirds of the participants (66.3%) never heard of HPV vaccines. Also, a high proportion of the participants were either unsure or stated that HPV vaccine was not registered in their vaccine schedule (45.3 and 37%, respectively). Regarding the participants’ knowledge of who should receive HPV vaccine, most participants reported that girls and women should receive it (56 and 51.3%, respectively). Nevertheless, 28.8% of the participants express uncertainty about who should receive HPV vaccine.

**Table 4:** Participants’ knowledge of human papilloma virus vaccine (*n* = 386).

Question or item	*N* (%)
Have you heard of HPV vaccine?	
Yes	130 (33.7)
No	256 (66.3)
Is HPV vaccine registered in your vaccine schedule?^*^	
No	143 (37)
Unsure	175 (45.3)
For girls only	43 (11.1)
For both genders	25 (6.5)
Recommended age for HPV vaccine (years; mean ± SD)	6.74 ± 8.37
Who should receive HPV vaccine?^*^	
Women	216 (56)
Girls	198 (51.3)
Men	112 (29)
Boys	113 (29.3)
Unsure	111 (28.8)
Is HPV vaccine effective?	
Strongly agree	55 (14.2)
Agree	114 (29.5)
Disagree	7 (1.8)
Strongly disagree	4 (1)
I do not know	206 (53.4)
Are benefits of HPV vaccine more than the risks?	
Strongly agree	60 (15.5)
Agree	100 (25.9)
Disagree	15 (3.9)
Strongly disagree	8 (2.1)
I do not know	203 (52.6)
Unsure	

HIV, human immunodeficiency virus; HPV, human papilloma virus.

*Some participants selected more than one answer.

Overall, 43.7% of the participants showed a certain level of agreement on HPV vaccine effectiveness. Moreover, 41.4% of the participants demonstrated a good level of agreement that the benefits of HPV vaccine uptake outweigh the risk.

### 3.5 Participants’ acceptability of HPV vaccine uptake by their children

Overall, a large proportion of the participants demonstrated a good level of agreement regarding the necessity of HPV vaccination for both girls and boys (61.4 and 40.7%, respectively) as shown in Table 5. Furthermore, only 19.9% indicated that the vaccine was recommended by their child’s pediatrician. The key motivators for child vaccination against HPV included the desire to protect them from sexually transmitted diseases (34.7%), from cancer and/or genital warts (28.5%), and to protect future spouses (25.1%). Conversely, reasons for not vaccinating the child against HPV included lack of information (15.3%) and concerns regarding potential adverse effects (13.7%).

**Table 5:** Participants’ acceptability of human papilloma virus vaccine uptake by their children (*n* = 386).

Question or item	*N* (%)
Is HPV vaccine mandatory for girls?	
Strongly agree	104 (26.9)
Agree	133 (34.5)
Disagree	19 (4.9)
Strongly disagree	3 (0.8)
I do not know	127 (32.9)
Is HPV vaccine s mandatory for boys?	
Strongly agree	68 (17.6)
Agree	89 (23.1)
Disagree	56 (14.5)
Strongly disagree	9 (2.3)
I do not know	164 (42.5)
My child’s doctor recommended HPV vaccine	
Yes	77 (19.9)
No	116 (30.1)
I do not know	193 (50)
I will vaccinate my child	
Strongly agree	57 (14.8)
Agree	159 (41.2)
Disagree	67 (17.4)
Strongly disagree	9 (2.3)
I do not know	94 (24.4)
Why will you give HPV vaccine for your child?^*^	
To protect from STD	134 (34.7)
To protect from genital warts/cancer	110 (28.5)
To protect future spouse	97 (25.1)
Adherence to immunization schedule	52 (13.5)
Physician’s recommendation	32 (8.3)
Aware of cancer complications	21 (5.4)
Why would not you give HPV vaccine to your child?^*^	
I do not have enough information	59 (15.3)
Afraid of the side effects	53 (13.7)
I’d rather wait on my decision	13 (3.4)
My child is not at risk	18 (4.7)
I prefer that my child decides for herself/himself	11 (2.8)
Too many vaccines are given	11 (2.8)
My child is too young	12 (3.1)
What information do you require to vaccinate your child? ^*^	
Specific information about the vaccine safety	78 (20.2)
General information about HPV	67 (17.4)
Specific information about vaccine efficacy	64 (16.6)
General information about HPV vaccine	58 (15)
Recommendation from the physician	56 (14.5)
Where would you get information if you wanted to know about HPV?^*^	
Social media and internet	186 (48.2)
Pediatrician	133 (34.5)
Family medicine physician	158 (40.9)
Gynecologist	127 (32.9)
Infectious disease physician	127 (32.9)
Other healthcare provider	77 (19.9)
Urologist	59 (15.3)
Pharmacist	51 (13.2)
Ministry of Health advertisement	39 (10.1)
Family Friends	28 (7.3)
TV or radio	29 (7.5)
Child’s school	21 (5.4)
Newspaper or magazine	19 (4.9)
Nurse	16 (4.1)

HPV, human papilloma virus; STD, sexually transmitted disease.

*Some participants selected more than one answer.

When considering what information that might persuade parents who were initially opposed to HPV vaccination for their child, the following needs were identified: 20.2% wanted details on vaccine safety, 16.6% requested information about the effectiveness of the HPV vaccine, 14.5% sought a recommendation from a physician, 17.4% wanted to learn more about HPV infection itself, and 15% were interested in general information about the HPV vaccine (Table 5). Consequently, about half of the participants (48.2%) stated that they would seek additional information about the HPV vaccine from social media and the internet, while 34.5% mentioned that they would consult a pediatrician for further information.

### 3.6 Factors associated with HPV vaccine uptake acceptability

Table 6 shows the results of the multivariable logistic regression to analyze the variables associated with HPV vaccine uptake acceptability. Having two or more children was significantly associated with a lower odds of vaccine acceptance (OR, 0.45; 95% CI, 0.23–0.86; *p* = 0.015), whereas the perception of HPV infection risk to other children if one‘s child is unvaccinated was associated with more than two odds of HPV vaccine acceptability (OR, 2.49; 95% CI, 1.37–4.52; *p* = 0.003). The remaining assessed factors did not show any association with HPV vaccine acceptability.

**Table 6:** Determinants of human papilloma virus vaccine uptake acceptability.

Factor	Odds ratio	95% Confidence interval	*P-*value
Relationship			0.715
Father	1.12	0.62–2.03	
Mother	Ref	Ref	
Age			0.251
<29–39	1.35	0.81–2.24	
≥40	Ref	Ref	
Education			0.231
School	Ref	Ref	
College	0.72	0.43–1.23	
Employment status			0.461
Employed	0.79	0.42–1.48	
Unemployed	Ref	Ref	
Social status			0.796
Married	1.14	0.43–3.03	
Other statuses	Ref	Ref	
Number of children			0.015
1	Ref	Ref	
≥2	0.45	0.23–0.86	
Child gender			0.200
Male	Ref	Ref	
Female	1.34	0.86–2.09	
Child age	1.03	0.99–1.08	0.182
Heard of HPV	1.52	0.92–2.52	0.099
Perception of HPV danger	1.35	0.85–2.13	0.209
Perception of vaccines effectiveness	1.09	0.51–2.30	0.831
Perception of vaccines harmfulness	0.55	0.24–1.26	0.154
Perception of risk to others if unvaccinated	2.49	1.37–4.52	0.003

HPV, human papilloma virus.

## 4 Discussion

The present study sought to assess the knowledge and attitudes of parents regarding the HPV vaccine in Saudi Arabia. Major findings revealed significant gaps in awareness and hesitancy toward vaccine uptake, where only 8.5% of the surveyed population’s children received the HPV vaccine. Such a small percentage reflects a barrier to prevent HPV-related cancer. Though, such a finding should be interpreted carefully given the study’s limitations discussed later in this section.

Our results align with the results of multiple studies conducted in different regions of Saudi Arabia, which reported similar trends of limited knowledge, low vaccination rates, and substantial barriers to vaccine acceptance. A recent study by AlShamlan et al. (25) reported that 20% of female healthcare workers (HCWs) received at least one dose of the HPV vaccine, with an additional 45% expressing willingness to receive it. While these figures indicate some level of acceptance among HCWs, they also highlight a significant knowledge gap; the HCWs had similar misconceptions about the vaccine that mirror those observed among parents in our study. In both cases, awareness was correlated with educational attainment, suggesting that increasing educational outreach can be a pivotal strategy for improving vaccine uptake (25). Another study by Alosaimi et al. (27) also found that a longer medical experience, especially in immunizations, was associated with odds more than 5 times of having better knowledge and a more positive attitude toward the HPV vaccine. Furthermore, a study from the western region of Saudi Arabia demonstrated that only 34.6% of women were aware of HPV, and awareness was higher among younger participants (22). Our findings echoed this trend, with older parents showing lower vaccination acceptance rates and a tendency toward risk minimization. Both studies suggest that younger aged participants are more informed about HPV and its vaccine, highlighting the need for targeted educational initiatives aimed at older parents who may be less engaged in health-related topics. In another study, it was found that only 32.9% of parents knew about the HPV vaccine, with a staggering 75.2% believing they were not at risk (30). This perception of risk is a substantial barrier to vaccination, as reflected in our findings, where many parents expressed uncertainty or hesitancy. The alignment of these results underscores a pressing need to address misconceptions surrounding HPV and its associated health risks in order to foster a more informed and proactive attitude toward vaccination. Despite the positive attitude toward vaccination among many parents in our study, where 60% expressed willingness to vaccinate, the actual uptake was significantly low, akin to findings from other studies. For instance, in the study by AlShamlan et al. (25), while there was a willingness among HCWs, actual vaccination rates remained low, revealing a disconnect between intention and action. This highlights the necessity of developing intervention strategies that not only educate parents about HPV and the vaccine but also facilitate the vaccination process through school-based programs and community initiatives.

The findings on awareness and understanding of HPV among participants revealed a significant gap in public knowledge. A majority (63.9%) reported being unaware of HPV, consistent with another study conducted in Saudi Arabia, where more than half of the participants (51%) were also uninformed about the virus (23). Moreover, the main source of information about HPV reported by participants in our study was the internet and social media, which is similar to previous studies from Saudi Arabia, which found that 33–73.7% of participants used social media as their source of information (23, 26, 31). Additionally, our results indicated a lack of understanding regarding how HPV is transmitted. Over half of the participants were uncertain about its transmission routes, with only 32.5% identifying sexual contact as a means of transmission. The low percentage suggesting public toilets as a transmission route highlights misconceptions that should be clarified through targeted educational efforts. This lack of knowledge of HPV could be attributed to the source of information (internet and social media), which may not have been credible resources. Though, many participants perceived HPV as a potentially dangerous disease. Less than half of those unaware of HPV had sufficient information to assess its severity, yet nearly half believed it to be dangerous. Furthermore, less than half of the participants (49.5%) expressed being uninformed about diseases associated with HPV, with only 32.4% recognizing cervical cancer as a related disease. In contrast, a study conducted in Switzerland found that while most participants primarily associated HPV with cervical cancer, many were unaware that it can also lead to other cancers, such as anal and oropharyngeal cancers (32). This gap in knowledge underscores the necessity for comprehensive educational efforts to illuminate the connections between HPV and various health outcomes.

Vaccination against HPV has been shown to be the single most effective method to prevent acquiring the virus. Yet public acceptance of the HPV vaccination is yet to be achieved in Saudi Arabia. The majority of respondents acknowledged the effectiveness of vaccines, yet a few were uncertain. Our study showed that 48.2% of participants primarily use the internet and social media for information, consistent with another study from Riyadh in the central region (31). While these platforms provide valuable resources, they also risk spreading misinformation and reinforcing negative beliefs, emphasizing the importance of accurate communication in public health initiatives. Similarly, the impact of misinformation on social media is evident in our findings, where 7.5% of respondents believed vaccines could be harmful. This mirrors findings from the perception of the public regarding COVID-19 vaccines who mostly obtained their information from social media (33). These observations confirm that anti-vaccine content on social media plays an influential role in shaping negative beliefs regarding the risk of vaccination. To combat misconceptions, it is crucial to use reliable online sources like the Saudi Ministry of Health’s social media and website. Promoting digital literacy and critical evaluation of online content is essential for identifying credible information. On the other hand, a study from Spain revealed that 92.1% of parents were aware of the HPV vaccine, with 62.3% relying on pediatricians for information (28). This reliance on credible healthcare professionals over social media suggests that effective education can improve awareness and acceptance, ultimately boosting vaccination rates.

Among the participants in our study, only one-third (33.7%) have heard of the HPV vaccine, demonstrating low awareness. Previous studies from Saudi Arabia demonstrated an awareness level of the vaccine in the range of 10.9–45.8% (10, 22–24, 30, 34). The difference in awareness between these populations may reflect the variability in public health campaigns and the dissemination of HPV vaccine information. Nonetheless, more than half of the participants in our study said that women and girls should receive the vaccine (56 and 51.3%, respectively) vs. about one-third stating the same for men and boys (29 and 29.3%, respectively). This observation was reflected in the answers on the question pertaining to the conditions that could be prevented by HPV vaccine, where cervical cancer was the most selected answer, though at a small rate of 32.4%. Similarly, a study by Akkour et al. (10) revealed that only 18.1% of women in Saudi Arabia were aware of the role of HPV vaccine in preventing cervical cancer.

The findings of this study offer valuable insights into parental acceptability and knowledge about the HPV vaccine. A particularly noteworthy observation is that a significant proportion of parents (84.5%) reported not getting vaccinated against HPV. This low uptake is likely multifactorial. A primary contributing factor appears to be a substantial lack of awareness, as evidenced by over two-thirds of our study’s population reporting no prior knowledge of the HPV vaccine. Furthermore, the cultural stigma associated with HPV infection as a sexually transmitted disease within the conservative cultural context of Saudi Arabia may also significantly contribute to vaccine hesitancy and a lack of interest in uptake. Further research is warranted to elucidate the relative contributions of these factors and to develop targeted public health interventions. Moreover, numerous participants were unaware of the inclusion of the HPV vaccine in the Ministry of Health’s childhood vaccine schedule of girls. This may be attributed to the fact that the vaccine was incorporated into the Saudi immunization schedule only recently in 2019 (18). Moreover, our results indicate that the acceptability of the HPV vaccine among parents is a mere 56%, reflecting a concerning level of hesitance despite the reported perception of the vaccine’s benefit over its risk was expressed by less than half of the respondents (41.4%). These low values raise significant doubts about the willingness to support vaccination efforts for both girls and boys, suggesting substantial barriers for public health initiatives aimed at enhancing awareness and understanding of the vaccine’s benefits. Our findings correspond with other studies concerning HPV vaccine awareness and acceptance, which reveal a troubling trend. A recent study conducted in the Eastern Province of Saudi Arabia indicated that only 47.7% of participants expressed a willingness to receive the HPV vaccine (23), while a study from Jazan in the southern region reported a slightly better, yet still inadequate, acceptability rate of 53% (35). Furthermore, another study focusing on parents of daughters in Riyadh (central region) revealed a somewhat higher acceptance rate of 60% (31); however, this still indicates that a considerable portion of parents remains either opposed to or indifferent regarding vaccination. In contrast, studies from Turkey, Africa, Thailand, and Spain revealed a much higher parental acceptance rate of 70, 79.5, 85, and 86.1%, respectively (28, 36–38). However, in Japan, the acceptance rate was only 21.8% (39). Collectively, these values underscore the significant disparity and the urgent need for focused interventions in Saudi Arabia to enhance vaccine acceptance.

The role of healthcare providers in recommending the HPV vaccine is crucial, as our findings showed that 15.8% of the participants heard of HPV from different healthcare providers. Awareness offered by healthcare providers can significantly influence parental decisions regarding their children’s health. However, it is concerning that 50% of parents were uncertain about whether their child’s physician recommends the HPV vaccine. This gap in communication highlights the need for improved dialog between healthcare providers and parents, as clear recommendations from physicians could enhance awareness and acceptance of the HPV vaccine, leading to higher vaccination rates and better public health outcomes.

While some parents expressed hesitancy to vaccinate their children due to fears of side effects and lack of information, this underscores the need for educational resources to clarify the HPV vaccine’s safety and efficacy. In contrast, a study in the Eastern Province of Saudi Arabia found that 48% of parents felt protected, with 38% citing information gaps as their reason for hesitancy (23). Similarly, 38% of parents in Jazan (southern Saudi Arabia) were hesitant due to perceptions of the vaccine being new (35). These regional differences highlight the necessity for tailored educational interventions to effectively address specific concerns.

Regarding the factors associated with vaccine uptake acceptability, we noted that only parents of two or more children tended to be hesitant to accept the vaccine, whereas the perception of HPV infection risk to others was positively associated with vaccine acceptability. This indicate that spreading the awareness of HPV risks and its oncogenic nature may enhance the acceptability of the vaccine among the public. The reason behind the lack of vaccine acceptance among parents of more than two children is unknown, but it is probably due to the burden placed on the parents to vaccinate multiple children and keep up with the childhood vaccines schedule. Nevertheless, the availability of numerous immunization clinics in urban and rural regions of Saudi Arabia should allow an easy access to vaccinations. Of note, other factors including parent’s gender, parent’s age, child gender, level of education and employment, having heard of HPV, perception of HPV danger, and perception of vaccine’s effectiveness and harmfulness were not significantly associated with disposition toward HPV vaccination. This contrasts with a study conducted on nurses in the United State, where the belief in efficacy of the HPV vaccine strongly predicted acceptance of the vaccine (40). Additionally, a study by Fallatah et al. (26) found that a better knowledge of HPV infection and risk of cervical cancer was significantly associated with a positive attitude regarding HPV vaccine uptake. Notably, the gender disparity in our study, where fathers outnumbered the mothers, did not impact the attitude toward HPV vaccine. Such observation was also seen in previous studies from Saudi Arabia where no significant difference was observed in terms of both HPV knowledge and attitude toward HPV vaccine between males and females (26, 27).

While this study provides some useful insights into the knowledge of the public in Saudi Arabia regarding HPV and its vaccine, as well as the willingness to vaccinate children, some limitations should be acknowledged. Some eligible people were hesitant to participate and thus refused to take part in the study, which led to the extension of the data collection period to secure a sufficient number of participants to meet the required sample size. Furthermore, the utilization of purposive sampling method was another contributing factor to the extension of the data collection period to ensure surveying only the targeted population. It may also limit the generalizability of the results to future parents, as well as to other regions of Saudi Arabia as interviewing with participants was mainly done in one city; though, many participants reported coming from different cities of the country. Recall bias is another important limitation, where participants may have had difficulty remembering certain information regarding HPV knowledge and vaccine uptake history and recommendations by the child’s physician. Moreover, the expression of socially acceptable answers is typically expected in survey-based studies, which represent social desirability bias. These two forms of bias may have an impact on the reliability of the results, which should be interpreted carefully. Additionally, the response rate could not be calculated due to the lack of a defined sampling pool as sampling was done randomly though only with a targeted population. Lastly, we did not collect data on socioeconomic status of the participants to assess the impact of such a factor on HPV vaccine acceptability. We also did not analyze the impact of regional differences among the participants since a reasonable proportion of the study’s population were non-Saudis and because data collection took place in only one city.

## 5 Conclusion

Results from this study indicated an overall lack of knowledge of the public in Saudi Arabia regarding HPV infection and its associated cancer risks, which may have been associated with a reduced willingness to vaccinate their children. Healthcare providers are encouraged to educate their patients and the public about HPV and the importance of the vaccine in media outlets and in their areas of practice.

## Data Availability

The raw data supporting the conclusions of this article will be made available by the authors, without undue reservation.
