# Supplementary material for: Knowledge, attitude, and perception of the parents toward HPV vaccine administration to their children in Saudi Arabia: a cross-sectional study
Source: Front Public Health. 2025 Jul 16;13:1531517. doi: 10.3389/fpubh.2025.1531517 (PMC12307288; doi:10.3389/fpubh.2025.1531517)
Supplement: Supplementary file 1 [file Data_Sheet_1.pdf]

English version of the questionnaire.

Part 1. Sociodemographic characteristics

1.1. Please indicate your relationship with the child you are answering for	Father<br>Mother<br>Other
1.2. Please indicate your age	Younger than 29 y.o<br>30–39 y.o<br>40–49 y.o<br>50–59 y.o<br>60 or older y.o
1.3. Please indicate your highest level of education	No formal schooling<br>Elementary<br>Middle school<br>High school<br>Undergraduate degree<br>Master's degree/ PhD
1.4. Please indicate your current employment status	Unemployed<br>Employed<br>Housewife<br>Retired/pensioner<br>Student
1.5. Nationality	Saudi<br>Non-Saudi
1.6. Please indicate your social status	Married<br>Divorced<br>Widowed
1.7. How many children (biological/ adopted) do you have?	1<br>2<br>3<br>4 or more
1.8. Please indicate the gender of the child about you are answering this questionnaire	Male<br>Female
1.9. Please indicate the age of the child you are answering this questionnaire	… years
1.10. Referring to the child you are answering this questionnaire, is	Yes<br>No

he/she vaccinated against human papillomavirus (HPV)?	I'm not sure
1.11. Are you vaccinated against HPV?	Yes<br>No<br>I'm not sure

Part 2. Knowledge of HPV

2.1. Have you ever heard about HPV?	Yes<br>No *(If "No", answer directly question 2.3)*
2.2. Where does the information you have about HPV come from? *(please indicate all that apply)*	Pediatrician<br>Family doctor<br>Gynecologist<br>Urologist<br>Infectious disease doctor<br>Nurse<br>Pharmacist<br>Other health care professionals<br>School<br>Friends/relatives<br>Newspapers<br>TV/Radio<br>Internet/ social media<br>Others
2.3. In your opinion, how do you think HPV infection is transmitted? *(please indicate all that apply)*	Sexual transmission<br>Blood transfusion<br>Use of public toilets<br>During pregnancy (mother-to-child transmission)<br>I'm not sure

Please indicate the level of your agreement with the following statements:

	Strongly disagree	Disagree	Agree	Strongly agree	I don´t know
2.4. HPV is the most common sexually transmitted disease					
2.5. HPV is a dangerous disease					

2.6. In your opinion, who could get infected with HPV? *(please indicate all that apply)*	Girls<br>Women<br>Boys<br>Men<br>Nobody

	I'm not sure
2.7. Which diseases do you think are related to HPV? *(please indicate all that apply)*	Bladder cancer<br>Genital warts<br>HIV/AIDS<br>Hepatitis<br>Infertility<br>Penis cancer<br>Irritable bowel syndrome<br>Cancer of the oral cavity<br>Esophagus cancer<br>Recurrent cystitis<br>Anal cancer<br>Vulvar cancer<br>Vaginal cancer<br>Cervical cancer<br>None of the above<br>I'm not sure
2.8. In your opinion, which measures can be taken to prevent the transmission of HPV infection? *(please, indicate all that apply)*	Use of condoms<br>Vaccination against HPV<br>Late start of sexual relations<br>Good personal hygiene<br>Low sexual activity<br>Antibiotics<br>Birth control pill<br>It can't be prevented<br>I don't know

Part 3. General knowledge and acceptability of vaccines

Please indicate if you agree with the following statements:

	Strongly disagree	Disagree	Agree	Strongly agree	I do not have enough information to answer
3.1. Vaccination is an effective measure to prevent infectious diseases in children and adults					
3.2. Benefits thanks to vaccination are greater than risks					
3.3. Vaccination is harmful					

3.4. Parents not vaccinating their children put other people at risk					
3.5. I'm afraid of vaccinating my children					

Part 4. Knowledge of HPV vaccine

4.1. Have you ever heard about HPV vaccine?	Yes<br>No
4.2. Are you aware that HPV vaccination included in the childhood immunization schedule?	Yes, only for girls<br>Yes, for boys and girls<br>It is not included<br>I'm not sure
4.3. What is the recommended age for HPV vaccine?	… years<br>I'm not sure
4.4. In your opinion, who should receive HPV vaccine? *(please, indicate all that apply)*	Girls<br>Women<br>Boys<br>Men<br>Nobody<br>I'm not sure

Please indicate the level of agreement with the following statements:

	Strongly disagree	Disagree	Agree	Strongly agree	I do not have enough information to answer
4.6. HPV vaccine is effective					
4.7. The benefits of the HPV vaccine outweigh the risks					

Part 5. Acceptability of HPV vaccine

Please indicate if you agree with the following statements:

	Strongly disagree	Disagree	Agree	Strongly agree	I do not have enough information to answer

5.1. HPV vaccination in girls is necessary					
5.2. HPV vaccine in boys is necessary					
5.3. My child's doctor recommends the HPV vaccine to my child					
[Only if "No" or "I'm not sure" in 1.14] 5.4. I would vaccinate my son/daughter against HPV					

5.5. In case your answer to question 5.4 was "agree" or "fully agree", could you please indicate which are the main reasons why you would vaccinate your son/daughter against HPV? *(please, indicate all that apply)*	To protect them against sexually transmitted diseases<br>To protect them against genital cancer and/or genital warts<br>To protect their future sexual partners against genital cancer and/or genital warts<br>I am aware of cancer and its prevention, because I know some cases amongst my closest relatives and friends.<br>To comply with the immunization schedule<br>It was the doctor's recommendation
5.6. In case your answer to question 5.4 was "disagree" or "fully disagree", could you please indicate which are the main reasons why you would hesitate/refuse to vaccinate your son/daughter against HPV? *(please, indicate all that apply)*	I'm afraid of possible adverse effects<br>In my opinion, too many vaccines are given<br>My son/daughter is too young to be vaccinated<br>It is too late, my son/daughter has already had his/her first sexual intercourse<br>I don´t consider my son/daughter at risk of being infected with HPV<br>I prefer that my son/daughter decides later by him/herself to get vaccinated<br>My doctor told me vaccination is not necessary<br>I don't have enough information about HPV vaccine<br>Vaccine price<br>I'd rather wait before taking this decision<br>There isn't enough information about HPV vaccination yet<br>Other
5.7. In case your answer to question 5.4 was "disagree" or "fully disagree", what type of information would you need to decide on vaccinating your son/daughter against HPV? *(please, indicate all that apply)*	Specific information about vaccine safety<br>Specific information about vaccine efficacy<br>General information about HPV<br>General information about HPV vaccine<br>Recommendation from my Doctor<br>Other
5.8. Who/where would you consult to obtain more information about HPV	Pediatrician<br>Family doctor<br>Gynecologist

vaccine? *(please, indicate all that apply)*	Urologist<br>Infectious disease doctor<br>Nurse<br>Pharmacist<br>Other health care professionals<br>Your child's school<br>Friends/relatives<br>Newspapers<br>TV/Radio<br>Internet/social media<br>Ministry of Health
