# Supplementary material for: Knowledge, attitude, and perception of the parents toward HPV vaccine administration to their children in Saudi Arabia: a cross-sectional study
Source: Front Public Health. 2025 Jul 16;13:1531517. doi: 10.3389/fpubh.2025.1531517 (PMC12307288; doi:10.3389/fpubh.2025.1531517)
Supplement: Supplementary file 2 [file Data_Sheet_2.pdf]

استبيان المعرفة بلقاح فيروس الورم الحليمي البشري وسلوك الآباء والأمهات تجاه إعطائه لبناتهم وأبنائهم

1. 1.1 صلة القرابة بالطفل :

Mark only one oval.

- أب
- أم
- Other: ____________________

2. 1.2 العمر :

Mark only one oval.

- اقل من 29 سنة
- 30-39
- 40-49
- 50-59
- 60 او اكبر

3. 1.3 المستوى التعليمي

Mark only one oval.

- أمي/ة
- ابتدائي
- متوسط
- ثانوي
- بكالوريس
- دراسات عليا

4. 1.4 الحالة الوظيفة

Mark only one oval.

- طالب /ة
- موظف /ة دوام جزئي
- موظف /ة دوام كامل
- متقاعد/ة
- عجز مؤقت

5. 1.5 مكان الاقامة

____

6. 1.6 مكان الميلاد

Mark only one oval.

- ( ) السعودية
- ( ) Other: ______________________

7. 1.7 الجنسية

Mark only one oval.

- ( ) السعودية
- ( ) Other: ______________________

8. 1.8اذا كنت غير سعودي/ة حدد مدة اقامتك في السعودية ض

Mark only one oval.

- ( ) أقل من 12 شهر
- ( ) أكثر من 12 شهر

9. 1.9 الحالة الاجتماعية

Mark only one oval.

- ( ) متزوج/ة
- ( ) مطلق/ة
- ( ) ارمل/ة

10. 1.10 عدد الابناء

Mark only one oval.

- ( ) 1
- ( ) 2
- ( ) 3
- ( ) 4 او اكثر

11. 1.11 عمر وجنس الابناء

Check all that apply.

- [ ] الطفل الاول
- [ ] الطفل الثاني
- [ ] الطفل الثالث
- [ ] الطفل الرابع
- [ ] الطفل الخامس
- [ ] الطفل السادس
- [ ] الطفل السابع
- [ ] الطفل الثامن
- [ ] الطفل التاسع
- [ ] الطفل العاشر

12. 1.12 جنس الطفل الذي تقوم بالاجابة عنه

Mark only one oval.

- ذكر
- انثى

13. 1.13 عمر الطفل

____

14. 1.14 هل حصل الطفل/ة على لقاح فيروس الورم الحليمي البشري

Mark only one oval.

- نعم
- لا
- ربما

15. 1.15 هل حصلت/ي على لقاح فيروس الورم الحليمي البشري

Mark only one oval.

- نعم
- لا
- ربما

16. 2.1 هل سمعت عن الورم الحليمي البشري

Mark only one oval.

- نعم
- لا، اذا كانت اجابتك بلا فانتقل لسؤال ال 2.3

17. 2.2 حصلت على معلوماتك حول الورم الحليمي البشري مين اين

Mark only one oval.

- طبيب الاطفال
- طبيب العائلة
- طبيب امراض
- طبيب المسالك البولية
- ممرض/ة
- صيدلاني/ة
- الاصدقاء /الاقارب
- الصحف /المجلات
- راديو /تلفزيون
- انترنت/ وسائل التواصل الاجتماعي
- Other: _______________________________

18. 2.3 كيف تنتقل عدوى فيروس الورم الحليمي البشري من وجهة نظرك

Mark only one oval.

- عن طريق الاتصال الجنسي
- عبر الدم
- عن طريق استخدام المراحيض العامة
- انتقال العدوى من الام الى الطفل
- لست متاكد/ة

19. حدد مدى موافقتك على العبارات التالية

Check all that apply.

	غير موافق تماما	غير موافق	موافق تماما	موافق	لا اعلم
**2.4 يعد فيروس الورم الحليمي البشري من اكثر الامراض الجنسية انتشارًا 1**	☐	☐	☐	☐	☐
**2.5 يعد فيروس الورم الحليمي البشري من الامراض الخطيرة**	☐	☐	☐	☐	☐

20. 2.6 كثر عرضة للإصابة بالورم الحليمي البشري من وجهة نظرك من هم الفئة الا

Mark only one oval.

- الفتيات
- النساء
- الفتيان
- الرجال
- لا احد
- غير متاكد

21. 2.7 بالورم الحليمي البشري من اعتقادكَارتباطاماهي الامراض الاكثر

Mark only one oval.

- سرطان المثانة
- الثآليل التناسلية
- فيروس نقص المناعة البشرية/ الايدز
- التهاب الكد الوبائي
- العقم
- سرطان القضيب
- متلازمة القولون المتهيج
- سرطان الف
- سرطان المري
- التهاب المثانة المتكررة
- سرطان الشرج
- سرطان الفرج
- سرطان المهبل
- سرطان عنق الرحم
- لا شيء مما ذكر
- لست متاكد

22. 2.8 التدابير التي يمكن اتخاذها لمنع انتقال عدوى فيروس الورم الحليمي البشري في رايك

Mark only one oval.

- استخدام الواقي الذكري
- التطعيم ضد فايروس الورم الحليمي البشري
- التاخر في بداية العلاقة الجنسية
- النظافة الشخصية الجيدة
- ضعف النشاط الجنسي
- المضادات الحيوية
- حبوب منع ال
- لا يمكن منعه
- لا اعلم

23. 2.9 ماهي مصادرك للحصول على معلومات إضافية حول فيروس الورم الحليمي البشري

Mark only one oval.

- طبيب الاطفال
- طبيب الاسرة
- طبيب امراض النساء
- طبيب المسالك البولية
- ممرض /ة
- صيدلاني/ة
- غيرهم من المتخصصين في الرعاية الصحية
- الاصدقاء / الاقارب
- الصحف / المجلات
- راديو / تلفزيون
- الانترنت / وسائل التواصل الاجتماعي
- Other: ____________

24. 3.1 التطعيم هو إجراء فعال للوقاية من الأمراض المعدية لدى الأطفال والبالغين

Mark only one oval.

- لا أوافق بشدة
- لا أوافق
- أوافق
- أوافق بشدة
- ليس لدي معلومات كافية للإجابة

25. 3.2 فوائد التطعيم أكثر من مخاطره

Mark only one oval.

- لا أوافق بشدة
- لا أوافق
- أوافق
- أوافق بشدة
- ليس لدي معلومات كافية للإجابة

26. ى3.3 التطعيم إجراء عديم الفائدة

Mark only one oval.

- لا أوافق بشدة
- لا أوافق
- أوافق
- أوافق بشدة
- ليس لدي معلومات كافية للإجابة

27. 3.4 عدم قيام الآباء بتطعيم أطفالهم يعرض الآخرين للخطر

Mark only one oval.

- لا أوافق بشدة
- لا أوافق
- أوافق
- أوافق بشدة
- ليس لدي معلومات كافية للإجابة

28. 3.5 أخاف من تطعيم أطفال

Mark only one oval.

- لا أوافق بشدة
- لا أوافق
- أوافق
- أوافق بشدة
- ليس لدي معلومات كافية للإجابة

29. 4.1 هل سمعت من قبل عن لقاح فيروس الورم الحليمي البشري

Mark only one oval.

- نعم
- لا

30. 4.2 من أين تأتي معلوماتك عن لقاح فيروس الورم الحليمي البشري؟ (يرجى الإشارة إلى كل ماينطبق)

Check all that apply.

- [ ] طبيب الأطفال
- [ ] طبيب العائلة
- [ ] طبيب أمراض النساء
- [ ] طبيب مسالك بولية
- [ ] ممرضة
- [ ] صيدلي
- [ ] غيرهم من المتخصصين في الرعاية الصحية
- [ ] مدرسة أطفال
- [ ] الأصدقاء/ الأقارب
- [ ] الصحف/ المجلات
- [ ] راديو/ تلفزيون
- [ ] الإنترنت/ وسائل التواصل الاجتماعي
- [ ] آخرون

31. 4.3 هل التطعيم ضد فيروس الورم الحليمي البشري مدرج في سجل التطعيمات في مجتمعك؟

Mark only one oval.

- نعم، للفتيات فقط
- نعم، للبنين والبنات
- لم يتم تضمينه
- لست متأكد/ متأكدة

32. 4.4 ماهو العمر الموصى به للقاح فيروس الورم الحليمي البشري؟

____

33. 4.5 في رأيك، من يمكنه تلقي لقاح فيروس الورم الحليمي البشري؟ (يرجى الإشارة إلى كل ما ينطبق)

Mark only one oval.

- فتيات
- نساء
- أولاد
- رجال
- لا أحد
- لست متأكد

34. 4.6 لقاح فيروس الورم الحليمي البشري فعال

Mark only one oval.

- لا أوافق بشدة
- لا أوافق
- أوافق
- أوافق بشدة
- ليس لدي معلومات كافية للإجابة

35. 4.7 فوائد لقاح فيروس الورم الحليمي البشري تفوق مخاطره

Mark only one oval.

- لا أوافق بشدة
- لا أوافق
- أوافق
- أوافق بشدة
- ليس لديمعلومات كافية للإجابة

36. 4.8 في رأيك، يمنع لقاح فيروس الورم الحليمي البشري (أشير إلى كل ما ينطبق):

Check all that apply.

- [ ] سرطان المثانة
- [ ] الثآليل التناسلية
- [ ] فيروس نقص المناعة البشرية / الإيدز
- [ ] التهاب الكبد
- [ ] العقم
- [ ] سرطان القضيب
- [ ] متلازمة القولون المتهيّج
- [ ] سرطان تجويف الفم
- [ ] سرطان المريء
- [ ] التهاب المثانة المتكرر
- [ ] سرطان الشرج
- [ ] سرطان الفرج
- [ ] سرطان المهبل
- [ ] سرطان عنق الرحم
- [ ] لا شيء مما بالأعلى
- [ ] لست متأكدة / متأكد

37. 5.1 التطعيم ضد فيروس الورم الحليمي البشري ضروري عند الفتيات

Mark only one oval.

- لا أوافق بشدة
- لا أوافق
- أوافق
- أوافق بشدة
- ليس لدي معلومات كافية للإجابة

38. 5.2 لقاح فيروس الورم الحليمي البشري عند الأولاد ضروري

Mark only one oval.

- لا أوافق بشدة
- لا أوافق
- أوافق
- أوافق بشدة
- ليس لدي معلومات كافية للإجابة

39. 5.3 يوصي الطبيب بلقاح فيروس الورم الحليمي البشري

Mark only one oval.

- لا أوافق بشدة
- لا أوافق
- أوافق
- أوافق بشدة
- ليس لدي معلومات كافية للإجابة

40. فقط إذا كانت الإجابة "لا" أو "لست متأكدًا" في 1.14
5.4 سأقوم بتطعيم ابني/ابنتي ضد فيروس الورم الحليمي البشري

Mark only one oval.

- لا أوافق بشدة
- لا أوافق
- أوافق
- أوافق بشدة
- ليس لدي معلومات مافية للإجابة

41. في حال كانت إجابتك على السؤال 5.3 "موافق" أو "أوافق بشدة"، هل يمكنك توضيح الأسباب الرئيسية التي تدفعك إلى تطعيم ابنك/ابنتك ضد فيروس الورم الحليمي 5.4 البشري؟ (يرجى الإشارة إلى كل ما ينطبق)

Check all that apply.

- [ ] لحمايتهم من الأمراض المنقولة جنسياً
- [ ] لحمايتهم من سرطان الأعضاء التناسلية و/أو الثآليل التناسلية
- [ ] لحماية شركائهم الجنسيين في المستقبل من سرطان الأعضاء التناسلية و/أو الثآليل التناسلية
- [ ] أنا على دراية بالسرطان وطرق الوقاية منه، لأنني أعرف بعض الحالات بين أقرب الأقارب والأصدقاء.
- [ ] الالتزام بجدول التحصينات
- [ ] لقد كانت توصية الطبيب

42. 5.5 في حال كانت إجابتك على السؤال 5.3 "لا أوافق" أو "لا أوافق بشدة"، هل يمكنك توضيح الأسباب الرئيسية التي تجعلك تتردد/ترفض تطعيم ابنك/ابنتك ضد فيروس الورم الحليمي البشري؟ (يرجى الإشارة إلى كل ما ينطبق)

Check all that apply.

- [ ] أخشى من الآثار السلبية المحتملة
- [ ] في رأيي، يتم إعطاء الكثير من اللقاحات
- [ ] ابني/ابنتي صغير جدًا بحيث لا يمكن تطعيمه
- [ ] لقد فات الأوان، فقد قام ابني/ابنتي بالفعل بممارسة أول علاقة جنسية
- [ ] لا أعتبر ابني/ابنتي معرضين لخطر الإصابة بفيروس الورم الحليمي البشري
- [ ] أفضّل أن يقرر ابني/ابنتي لاحقًا بنفسه الحصول على التطعيم
- [ ] أخبرني طبيبي أن التطعيم ليس ضروريًا
- [ ] ليس لدي معلومات كافية عن لقاح فيروس الورم الحليمي البشري
- [ ] سعر اللقاح
- [ ] أفضل الانتظار قبل اتخاذ هذا القرار
- [ ] لا توجد معلومات كافية حول التطعيم ضد فيروس الورم الحليمي البشري حتى الآن
- [ ] سبب آخر

43. 5.6 في حال كانت إجابتك على السؤال 5.3 "لا أوافق" أو "لا أوافق بشدة"، ما نوع المعلومات التي ستحتاجها لاتخاذ قرار بشأن تطعيم ابنك/ابنتك ضد فيروس الورم الحليمي البشري؟ (يرجى الإشارة إلى كل ما ينطبق)

Check all that apply.

- [ ] معلومات محددة حول سلامة اللقاحات
- [ ] معلومات محددة حول فعالية اللقاح
- [ ] معلومات عامة عن فيروس الورم الحليمي البشري
- [ ] معلومات عامة عن لقاح فيروس الورم الحليمي البشري
- [ ] توصية من طبيبي
- [ ] سبب آخر

44. 5.7 من/أين يمكنك التشاور للحصول على مزيد من المعلومات حول لقاح فيروس الورم الحليمي البشري؟ (يرجى الإشارة إلى كل ما ينطبق)

Check all that apply.

- [ ] طبيب الأطفال
- [ ] طبيب العائلة
- [ ] طبيب أمراض نساء
- [ ] طبيب مسالك بولية
- [ ] ممرضة
- [ ] صيدلي
- [ ] غيرهم من المتخصصين في الرعاية الصحية
- [ ] مدرسة طفلك
- [ ] الأصدقاء / الأقارب
- [ ] الصحف / المجلات
- [ ] راديو / تلفزيون
- [ ] الإنترنت/ وسائل التواصل الاجتماعي
- [ ] آخرون

45. 3.1 التطعيم هو إجراء فعال للوقاية من الأمراض المعدية لدى الأطفال والبالغين

Mark only one oval.

- ( ) لا أوافق بشدة
- ( ) لا أوافق
- ( ) أوافق
- ( ) أوافق بشدة
- ( ) ليس لدي معلومات كافية للإجابة

46. 3.2 فوائد التطعيم أكثر من مخاطره

Mark only one oval.

- لا أوافق بشدة
- لا أوافق
- أوافق
- أوافق بشدة
- ليس لدي معلومات كافية للإجابة

47. 3.3 التطعيم إجراء عديم الفائدة

Mark only one oval.

- لا أوافق بشدة
- لا أوافق
- أوافق
- أوافق بشدة
- ليس لدي معلومات كافية للإجابة

48. 3.4 عدم قيام الآباء بتطعيم أطفالهم يعرض الآخرين للخطر

Mark only one oval.

- لا أوافق بشدة
- لا أوافق
- أوافق
- أوافق بشدة
- ليس لدي معلومات كافية للإجابة

49. 3.5 أخاف من تطعيم أطفال

Mark only one oval.

- لا أوافق بشدة
- لا أوافق
- أوافق
- أوافق بشدة
- ليس لدي معلومات كافية للإجابة

50. 4.1 هل سمعت من قبل عن لقاح فيروس الورم الحليمي البشري

Mark only one oval.

- نعم
- لا

51. 4.2 من أين تأتي معلوماتك عن لقاح فيروس الورم الحليمي البشري؟ (يرجى الإشارة إلى كل ماينطبق)

Check all that apply.

- [ ] طبيب الأطفال
- [ ] طبيب العائلة
- [ ] طبيب أمراض النساء
- [ ] طبيب مسالك بولية
- [ ] ممرضة
- [ ] صيدلي
- [ ] غيرهم من المتخصصين في الرعاية الصحية
- [ ] مدرسة أطفال
- [ ] الأصدقاء/ الأقارب
- [ ] الصحف/ المجلات
- [ ] راديو/ تلفزيون
- [ ] الإنترنت/ وسائل التواصل الاجتماعي
- [ ] آخرون

52. 4.3 هل التطعيم ضد فيروس الورم الحليمي البشري مدرج في سجل التطعيمات في مجتمعك؟

Mark only one oval.

- نعم، للفتيات فقط
- نعم، للبنين والبنات
- لم يتم تضمينه
- لست متأكد/ متأكدة

53. 4.4 ماهو العمر الموصى به للقاح فيروس الورم الحليمي البشري؟

____

54. 4.5 في رأيك، من يمكنه تلقي لقاح فيروس الورم الحليمي البشري؟ (يرجى الإشارة إلى كل ما ينطبق)

Mark only one oval.

- فتيات
- نساء
- أولاد
- رجال
- لا أحد
- لست متأكد

55. 4.6 لقاح فيروس الورم الحليمي البشري فعال

Mark only one oval.

- لا أوافق بشدة
- لا أوافق
- أوافق
- أوافق بشدة
- ليس لدي معلومات كافية للإجابة

56. 4.7 فوائد لقاح فيروس الورم الحليمي البشري تفوق مخاطره

Mark only one oval.

- لا أوافق بشدة
- لا أوافق
- أوافق
- أوافق بشدة
- ليس لديمعلومات كافية للإجابة

57. 4.8 في رأيك، يمنع لقاح فيروس الورم الحليمي البشري (أشير إلى كل ما ينطبق):

Check all that apply.

- [ ] سرطان المثانة
- [ ] الثآليل التناسلية
- [ ] فيروس نقص المناعة البشرية / الإيدز
- [ ] التهاب الكبد
- [ ] العقم
- [ ] سرطان القضيب
- [ ] متلازمة القولون المتهيّج
- [ ] سرطان تجويف الفم
- [ ] سرطان المريء
- [ ] التهاب المثانة المتكرر
- [ ] سرطان الشرج
- [ ] سرطان الفرج
- [ ] سرطان المهبل
- [ ] سرطان عنق الرحم
- [ ] لا شيء مما بالأعلى
- [ ] لست متأكدة / متأكد

58. 5.1 التطعيم ضد فيروس الورم الحليمي البشري ضروري عند الفتيات

Mark only one oval.

- لا أوافق بشدة
- لا أوافق
- أوافق
- أوافق بشدة
- ليس لدي معلومات كافية للإجابة

59. 5.2 لقاح فيروس الورم الحليمي البشري عند الأولاد ضروري

Mark only one oval.

- لا أوافق بشدة
- لا أوافق
- أوافق
- أوافق بشدة
- ليس لدي معلومات كافية للإجابة

60. 5.3 يوصي الطبيب بلقاح فيروس الورم الحليمي البشري

Mark only one oval.

- لا أوافق بشدة
- لا أوافق
- أوافق
- أوافق بشدة
- ليس لدي معلومات كافية للإجابة

61. فقط إذا كانت الإجابة "لا" أو "لست متأكدًا" في 1.14
5.4 سأقوم بتطعيم ابني/ابنتي ضد فيروس الورم الحليمي البشري

Mark only one oval.

- لا أوافق بشدة
- لا أوافق
- أوافق
- أوافق بشدة
- ليس لدي معلومات مافية للإجابة

62. 5.4 في حال كانت إجابتك على السؤال 5.3 "موافق" أو "أوافق بشدة"، هل يمكنك توضيح الأسباب الرئيسية التي تدفعك إلى تطعيم ابنك/ابنتك ضد فيروس الورم الحليمي البشري؟ (يرجى الإشارة إلى كل ما ينطبق)

Check all that apply.

- لحمايتهم من الأمراض المنقولة جنسياً
- لحمايتهم من سرطان الأعضاء التناسلية و/أو الثآليل التناسلية
- لحماية شركائهم الجنسيين في المستقبل من سرطان الأعضاء التناسلية و/أو الثآليل التناسلية
- أنا على دراية بالسرطان وطرق الوقاية منه، لأنني أعرف بعض الحالات بين أقرب الأقارب والأصدقاء.
- الالتزام بجدول التحصينات
- لقد كانت توصية الطبيب

63. 5.5 في حال كانت إجابتك على السؤال 5.3 "لا أوافق" أو "لا أوافق بشدة"، هل يمكنك توضيح الأسباب الرئيسية التي تجعلك تتردد/ترفض تطعيم ابنك/ابنتك ضد فيروس الورم الحليمي البشري؟ (يرجى الإشارة إلى كل ما ينطبق)

Check all that apply.

- أخشى من الآثار السلبية المحتملة
- في رأيي، يتم إعطاء الكثير من اللقاحات
- ابني/ابنتي صغير جدًا بحيث لا يمكن تطعيمه
- لقد فات الأوان، فقد قام ابني/ابنتي بالفعل بممارسة أول علاقة جنسية
- لا أعتبر ابني/ابنتي معرضين لخطر الإصابة بفيروس الورم الحليمي البشري
- أفضّل أن يقرر ابني/ابنتي لاحقًا بنفسه الحصول على التطعيم
- أخبرني طبيبي أن التطعيم ليس ضروريًا
- ليس لدي معلومات كافية عن لقاح فيروس الورم الحليمي البشري
- سعر اللقاح
- أفضل الانتظار قبل اتخاذ هذا القرار
- لا توجد معلومات كافية حول التطعيم ضد فيروس الورم الحليمي البشري حتى الآن
- سبب آخر

64. 5.6 في حال كانت إجابتك على السؤال 5.3 "لا أوافق" أو "لا أوافق بشدة"، ما نوع المعلومات التي ستحتاجها لاتخاذ قرار بشأن تطعيم ابنك/ابنتك ضد فيروس الورم الحليمي البشري؟ (يرجى الإشارة إلى كل ما ينطبق)

Check all that apply.

- [ ] معلومات محددة حول سلامة اللقاحات
- [ ] معلومات محددة حول فعالية اللقاح
- [ ] معلومات عامة عن فيروس الورم الحليمي البشري
- [ ] معلومات عامة عن لقاح فيروس الورم الحليمي البشري
- [ ] توصية من طبيبي
- [ ] سبب آخر

65. 5.7 من/أين يمكنك التشاور للحصول على مزيد من المعلومات حول لقاح فيروس الورم الحليمي البشري؟ (يرجى الإشارة إلى كل ما ينطبق)

Check all that apply.

- [ ] طبيب الأطفال
- [ ] طبيب العائلة
- [ ] طبيب أمراض نساء
- [ ] طبيب مسالك بولية
- [ ] ممرضة
- [ ] صيدلي
- [ ] غيرهم من المتخصصين في الرعاية الصحية
- [ ] مدرسة طفلك
- [ ] الأصدقاء / الأقارب
- [ ] الصحف / المجلات
- [ ] راديو / تلفزيون
- [ ] الإنترنت/ وسائل التواصل الاجتماعي
- [ ] آخرون

This content is neither created nor endorsed by Google.

Google Forms
